# Is the Six Year Molar Less Perfect Than the Other Teeth?

**Published:** 1890-02

**Authors:** C. C. Merriman


### Is the Six Year Molar Less Perfect than the other Teeth? If so, in what Respect, and Why?

BY C. C. MERRIMAN.

Perfection refers to form and to structure. As to form, less imperfection can be found in the first molar than in any other tooth both as regards size and outline, being the largest tooth in the series, and as closely as any other conforming to its typical outline.

As regards structure, even our brief experience has shown us a marked inferiority to the other teeth in its power to resist disease. About two-thirds of all the teeth extracted in this college (U. of M. Dental Department) this year have been the six year molar. Dr. Taft places the proportion of decayed first molar at 37 per cent. of all decayed teeth. Analyzing tables given by Drs. Tomes, Hitchcock, and Magitot comprising about 35,000 cases of caries, find that we considerably over 25 per cent. of all decayed teeth are the six year molars.

In looking for the cause of this defective structure the first thing noticed influencing this tooth is its early eruption.

The body of a child is not the minature of an adult organism, but is characterized by peculiarities of structure and function. All its organs are incompletely developed but not uniformly so. The brain is, proportionately to size and weight, larger than in the adult, imperfect in structure and of softer consistency. The tissues generally are softer, more vascular, and more distended with fluids. The skeleton is, for the most part, cartilaginous, the muscles gelatinous, the cellular tissues are filled with serum, the skin vascular and sensitive. All the tissues at this period are in a formative condition, lacking strength and solidity, and characterized by the defects noticed in the first permanent tooth.

Deficient nutrition is that condition in which the quantity is inadequate to supply the natural demands of growth, the quality being normal. The result is a structure different in form or strength, but which, so far as is formed is of healthy appearance.

The cause of this deficiency, as far as the six year molar is concerned, is not that a less amount of nutrient material is in the system, rapidly formed and deposited; for the voracious appetite and vigorous digestion of the average six year old child would successfully dispute this, but that there is a demand made by every tissue in the young body for more than its share of nourishment, and the first molar becomes more and more incapable of receiving its supply, and once formed must take its chances much as it is, while the other tissues are developing powers of resistance and the means of reparation of injury.

Not only are all the tissues demanding nourishment, but especially its neighbors on either side are being developed and require supply which is had only at the expense of the first molar.

Anatomically considered no other portion of the human organism offers such a complex association of tissues as those of the mouth, no other has such diversified functions, and from a pathological standpoint no such significant systemic relations, and at the period at which the six year molar is developed the jaws are crowded with teeth, both of the temporary series and the partly grown permanent set; vital activity and irritability is at its maximum and the system generally, and mouth especially, susceptible of injury and disease. After its eruption it is surrounded by tissues irritated by the pressure of the teeth below, the gums easily irritated and inflamed, the temporary teeth being absorbed at their roots are becoming less firm in their attachment, and in every act of mastication aggravating the trouble, so that the tendency is to avoid using them depriving the new teeth of the exercise needed to strengthen and develop them.

Again, the tooth coming so early is popularly supposed to belong to the temporary set and its condition ignored till pain causes a visit to the dentist who relieves the patient of both pain and tooth. This cause of defect in this tooth is more important than it at first appears. Were it confined to a few it would be immaterial, but continued through generations, as it is, and multiplied by inherited tendency, any organ will succumb to such usage.

The treatment received by the teeth of a six year old child will account for much of their trouble. He does those things which he ought not to do and leaves undone those things which he ought to do. He will eat any thing that is sweet, and chew any thing he can call gum, but will not brush his teeth, nor as formerly, will he allow his nurse or mother to do it for him.

